# Preventing Sexual Harassment in Higher Education: A Framework for Prevention Science Program Development

**DOI:** 10.1007/s10935-024-00780-4

**Published:** 2024-04-13

**Authors:** Cindy A. Crusto, Lisa M. Hooper, Ishita S. Arora

**Affiliations:** 1 Office for Women in Medicine and Science, Office of Diversity, Equity and Inclusion, Yale University School of Medicine, New Haven, CT USA; 2 Center for Educational Transformation, University of Northern Iowa, Cedar Falls, Iowa USA

**Keywords:** Institutions of higher education, Sexual harassment, Prevention science, Program development, Prevention framework

## Abstract

Sexual harassment is an intractable problem that harms the students, community, culture, and success of institutes of higher education (IHEs). The alarming prevalence of sexual harassment at IHEs highlights the urgent need for effective prevention programs. However, there are few empirically supported preventive interventions that effectively target the factors that most impact the determinants, trajectory, and short- and intermediate-term effects of sexual harassment. In this paper, we overview the problem of sexual harassment and propose an organizing framework to help IHEs develop effective interventions to prevent sexual harassment. Guided by prevention science, we propose a framework—modified from SAMHSA’s (2019) guidelines for prevention practitioners—that underscores the criticality of trauma- and equity-informed characteristics in prevention programs. We offer a discussion on how IHEs must consider and evaluate the empirical evidence of effectiveness, flexibility, cultural competency, and sustainability when developing and adapting prevention programs to reduce and—ultimately—ameliorate sexual harassment. We conclude with recommendations that can provide a roadmap for higher education stakeholders and researchers to prevent this urgent public health concern.

## Introduction

Sexual harassment remains an intractable problem in institutions of higher education (IHEs) in the United States. Although sexual harassment is an epidemic and a public health problem, research detailing the prevalence of, the causes of, and empirically supported prevention-intervention strategies for sexual harassment remains inadequate (Basile et al., 2020; Bloom et al., 2021; Bonar et al., 2022; Dills et al., 2016). This lack of research limits institutional efforts to prevent sexual harassment. The purpose of this paper is three-fold: (a) to highlight the need for sexual harassment prevention (to reduce its prevalence, limit negative effects, and identify practical gaps), (b) to overview prevention science for higher education stakeholders (e.g., institutional leadership, faculty, community members), and (c) to provide an organizing framework to enable IHEs to implement effective sexual harassment prevention programs. We assert that prevention science principles are instrumental in key-decision making to identify, adopt, adapt, or develop sexual harassment prevention frameworks and programs within the IHE context. We contend that the comprehensive, evidence-based, and culturally sensitive nature of the proposed framework will aid in adopting, adapting or innovating prevention efforts with diverse stakeholders at IHEs (e.g., staff, students, faculty, and community members; Wong et al., 2017). We conclude with recommendations that can provide a roadmap for higher education stakeholders and researchers to prevent this urgent public health concern.

## Nature of the Problem

Sexual harassment is located on the spectrum of gender-based violence and discrimination and can range from gender slurs, sexist insults, and bullying to sexual assault or threatening professional consequences if sexual favors are unmet. Sexual harassment encompasses three categories of behavior: gender harassment, unwanted sexual attention, and sexual coercion (National Academies of Sciences, Engineering, and Medicine [NASEM], 2018). Gender-based harassment comprises of behaviors, both verbal and non-verbal, directed at members of one gender to convey “hostility, objectification, exclusion, or second-class status” (NASEM, 2018), including offensive remarks about bodies, insults to working mothers, unwanted sexual discussions, and more. Gender-based harassment, the most common form of sexual harassment (Bondestam & Lundqvist, 2020; Swedish Council of Higher Education [ACHE], 2020; Aycock et al., 2019), happens more often in environments that condone it. Unwanted sexual attention entails unwelcome sexual advances and sexual assault targeted at an individual, and sexual coercion occurs when favorable treatment towards an individual is a condition of their engagement in sexual activity.

The aftereffects of sexual harassment in IHEs can be pernicious and deleterious, leading to mental health challenges, substance use, decreased academic performance, impaired career trajectory, isolation, and helplessness for individuals (Marine & Hurtado, 2021). It also has negative impacts on workplaces, including substantial financial costs/damages; decreased employee motivation, satisfaction, and productivity; increased concerns about inequities; a hostile organizational climate; legal costs; and high personnel turnover (Bondestam & Lundqvist, 2020). A systematic analysis of research of students and staff at IHEs worldwide (1966–2017) shows that globally 11-73% of heterosexual women and 3-26% of heterosexual men in IHEs are exposed to sexual harassment, and these numbers are assumed to be far higher for people living with marginalized identities (e.g., age, race, ethnicity, sexuality, gender, dis/ability, immigration status, and prior victimization; Bondestam & Lundqvist, 2020). While approximately 45% of all students at IHEs in the U.S. experience sexual harassment, among the undergraduate students living with marginalized identities 31.3% of women and 46.3% of non-binary, transgender, and gender questioning individuals report experiencing sexual harassment (Cantor et al., 2019). Some scholars contend that the prevalence of sexual harassment at IHEs is significantly underestimated due to numerous factors (Burn, 2019; Cantor et al., 2019). These include underreporting due to stigma, sample size and heterogeneity, societal norms, legal context, and differences in research methodology such as conceptual frameworks, operational definitions, and measurement. Compared to other workplaces in the U.S., women in academia experience sexually harassing behaviors (58%) more than any other workplace except the military (69%; Ilies et al., 2003). Importantly, meta-analytical studies suggest that the prevalence of sexual harassment in IHEs has not declined over time (Bondestam & Lundqvist, 2020; Fnais et al., 2014).

### Limitations in Sexual Harassment Research and Practice

Most research on sexual and gender-based harassment is fraught with substantive limitations. The existing sexual harassment prevention frameworks tend to be more limited in scope than our proposed model. The existing models focus on sexual assault and violence (Dills et al., 2016) rather than the full spectrum of sexual harassment (and on short-term individual level outcomes rather than long-term individual behavioral change or system level change within IHEs (Bondestam & Lundqvist, 2020). Additionally, existing frameworks tend not to stress the evaluation of their efforts or the experiences of marginalized individuals, who tend to be at higher risk of sexual harassment (Guilbeau, et al. 2021; Coulter et al., 2017; Kafonek & Richards, 2017).

The existing gaps in sexual harassment prevention research and practice include, but are not limited to (a) data drawn from homogeneous student samples, (b) a focus on individualistic rather than systemic factors, (c) a lack of standardized measurement (d) a lack of understanding of risk and protective factors, (e) a lack of evidence of prevention programs and their effectiveness and sustainability, and (f) a lack of culturally responsive and trauma-informed implementation (McCauley & Casler, 2015). Additionally, most research conceptualizes and defines sexual harassment from a legal perspective and theorizes gender as simplistic and binary; these definitions are insufficient to grasp the full complexity of sexual harassment in IHEs. This insufficient theorization further constrains research questions and research methodology. For example, research on sexual harassment frequently fails to examine the underlying causal mechanisms of sexual harassment, the characteristics of the person causing the harm, and the people who are at the most risk of causing harm and/or experiencing sexual harassment (Anderson & Whiston, 2005; Vladutiu et al., 2011).

Similarly, several barriers hinder an effective institutional response to prevent and reduce sexual harassment at IHEs. These barriers include a lack of understanding of the root causes of the problem, overreliance on the idea that sexual harassment is an individual problem rather than an institutional problem, overreliance on fast fixes and simplistic solutions that fail to grapple with contextual depth and history of exclusion in academia, overemphasis on legal procedures, ill-informed and generic trainings, and lack of diverse leadership and stakeholders in the design of solutions to prevent sexual harassment (Bloom et al., 2021; Chambers et al., 2021; Clancy et al., 2020; Linder et al., 2020; Lisak & Miller, 2002). There is an urgent need to radically redesign sexual harassment prevention and response systems in IHEs (Clancy et al., 2020).

## Solution – Prevention Science: from Theory to Application

Prevention science can be effective in stopping or delaying sexual harassment from occurring with a specific focus on vulnerable populations, reducing the negative consequences of sexual harassment on a target community and promoting policies and practices to enhance well-being at the individual, organizational, and community levels (American Psychological Association [APA], 2014). The transdisciplinary science of prevention synthesizes empirical knowledge from the biopsychosocial sciences, including sociology, psychology, behavioral science, economics, medicine, epidemiology, and neurology. This synthesis approach can help determine the multi-level ecological conditions that lead to sexual harassment at IHEs and can help identify strategies, policies, procedures, and practices to reduce the incidence of sexual harassment (Bell et al., 2002). Prevention has a two-pronged goal – (a) to systematically study the “precursors of dysfunction or health, called risk factors and protective factors, respectively” (Coie et al., 1993, p. 1013), and (b) to develop, implement, and evaluate evidence-based practices that can decrease said risk factors and increase protective factors. Taken together, focusing on these two goals can reduce sexual harassment victimization and promote healthy higher education communities and organizations (Bell et al., 2002; Coie et al., 1993; Magley et al., 2013). In the context of higher education, Kafonek and Richards (2017) outlined the utility and transportability of six of nine principles of effective prevention programs (Nation et al., 2003) described below, toward reducing gender-based violence in higher education.

### Principles of Effective Prevention and a Proposed Needed Extension

Identifying underlying principles that guide prevention frameworks and programs can help in the successful development, adoption, adaptation of sustainable multi-level prevention strategies for sexual harassment that can transform the culture of IHEs. Bonar and colleagues (2022) emphasized that, “prevention from a public health perspective involves a set of coordinated multi-component strategies that address risk and protective factors across the social ecology, that complement and reinforce each other with consistent messaging from multiple sources across multiple contexts, including addressing the diverse student population” (p. 145–15). Rooted in these guidelines, six core principles of prevention science can inform the development and implementation of effective prevention programs (Nation et al., 2003). Research investigating the adherence of the six principles of prevention programs in diverse IHEs show that adherence to these principles is low in most IHEs and that this adherence often excludes a focus on perpetrators and on the populations that are at the highest risk of experiencing sexual harassment: racial, ethnic, gender, or sexual minority students, staff, and faculty (Kafonek & Richards, 2017).

Here, we incorporate nine characteristics of effective prevention programs introduced by Nation and colleagues (2003) and further expounded upon by Bonar and colleagues (2022) to propose a comprehensive list of principles for effective prevention targeting sexual harassment in IHEs (Table 1). Specifically, our recommendations add to previously established core principles by incorporating cultural competence, sustainability, and the trauma-informed and equity-informed nature of prevention and address limitations evinced in the literature about IHEs. *Cultural competence* is defined as “the ability of an individual or organization to understand and interact effectively with people who have different values, lifestyles, and traditions based on their distinctive heritage and social relationships” (Substance Abuse and Mental Health Services Administration [SAMHSA], 2019, p. 4). *Sustainability* is “the process of building an adaptive and effective system that achieves and maintains desired long-term results” (SAMHSA, 2019, p. 4). The trauma-informed nature of prevention focuses on the “contextual features of environments and institutions that give rise to trauma, maintain it, and impact posttraumatic responses” (Goldsmith et al., 2014, p. 118). *Trauma-informed* principles can include trauma-specific assessment, interventions and treatment, and structures supporting posttraumatic growth and recovery post-trauma. *Equity-informed* principles focus on mitigating system- and societal-level inequities that increase the risk of sexual harassment, such as historical disadvantage and structural inequalities (Shapiro et al., 2024).

**Table 1:** Principles for effective sexual harassment prevention programs (adapted from Nation et al., 2003)

Program Domains	Principles	Definition
Program characteristics	Comprehensive	Multicomponent interventions address critical domains (e.g., family, peers, community) that influence the development and perpetuation of the behaviors to be prevented
	Varied teaching methods	Programs involve diverse teaching methods that focus on increasing awareness and understanding of the problem behaviors and on acquiring or enhancing skills
	Dosage	Programs provide enough intervention to produce the desired effects and provide follow-up as necessary to maintain effects
	Theory driven	Programs have a theoretical justification, are based on accurate information, and are supported by empirical research
	Positive relationships	Programs provide exposure to adults and peers in a way that promotes strong relationships and supports positive outcomes
Program characteristics → target group/population	Appropriately timed	Programs are initiated early enough to have an impact on the development of the problem behavior and are sensitive to the developmental needs of participants
	Socio-culturally relevant	Programs are tailored to the community and cultural norms of the participants and make efforts to include the target group in program planning and implementation
Program → implementation	Outcome evaluation	Programs have clear goals and objectives and make an effort to systematically document their results relative to the goals
	Well-trained staff	Program staff support the program and are provided with training regarding the implementation of the intervention
Program characteristics and implementation**	Trauma-informed**	Program staff support are provided with training regarding how trauma may affect the development of risk and protective factors and outcomes
Program characteristics and implementation**	Equity-informed**	Program staff are provided with training regarding how inequities may have an impact on the development of risk and protective factors and outcomes

Note: ** = Adaptation appears in italics

SOURCE: Adapted from Nation et al. (2003)

### Ecological Systems Approach to Prevention

**Fig. 1:**
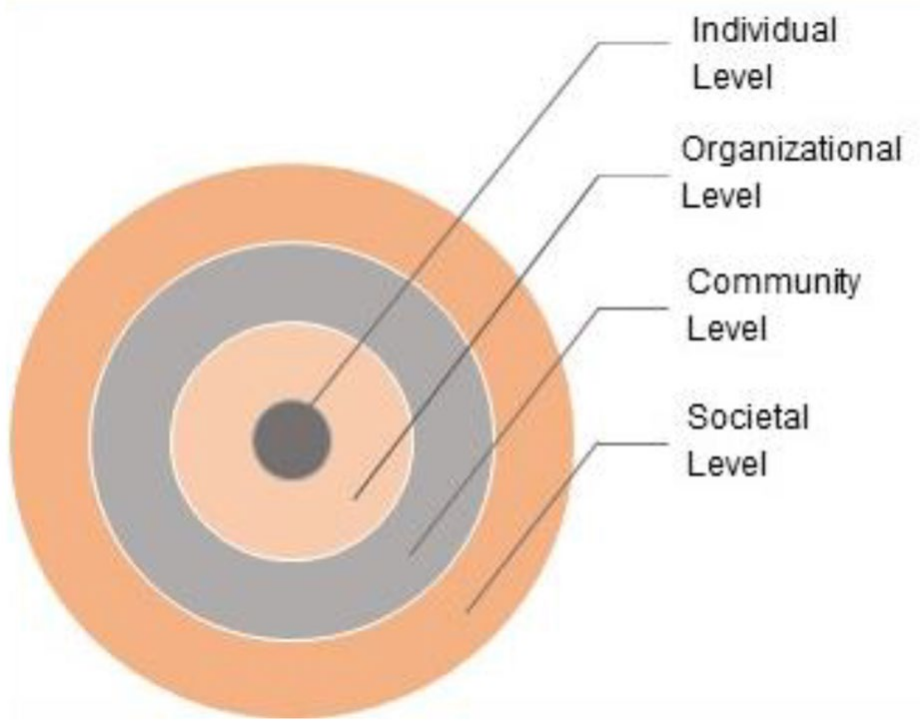
Ecological systems approach. SOURCE: Adapted from SAMHSA (2019)

Prevention efforts can be directed at multiple levels within the ecology of an IHE, either at each level individually or simultaneously across different levels of a system (see Fig. 1; individual, relational, organizational, community, and societal; APA, 2014; Dahlberg & Krug, 2002; Shapiro et al., 2024). For a sustained and meaningful reduction of sexual harassment, a multi-level, multi-pronged, and multi-determined approach to prevention is warranted (Clancy et al., 2020; Dills et al., 2016). The most effective way to achieve this is through an ecological systems approach which comprises of nested, overlapping and bidirectionally intersecting levels of the ecological system (Bronfenbrenner, 1976; Shapiro et al., 2024). Primary, secondary, and tertiary prevention strategies can target the varied risk and protective factors at each of the different levels simultaneously as part of an ecologically valid, culturally competent, and sustainable prevention framework. For such an approach to be effective, it is important to consider the unique ecological context of the IHE (e.g., comprehensive universities, Historically Black Colleges and Universities [HBCU], and community colleges) where the intended prevention program is being implemented by using the ecological systems approach (Fig. 1, adapted from SAMHSA, 2019), including identifying the community in which the IHE is embedded and assessing the needs, existing strengths, resources, and limitations of the IHE (Dills et al., 2016; DeGue et al., 2014).

### Multi-Level Risk and Protective Factors for Sexual Harassment in IHEs

Prevention programs can reduce or prevent sexual harassment by targeting empirically supported risk and protective factors at multiple levels of an IHE’s ecology. As the name denotes, risk factors are evidence-based variables/factors that increase the likelihood of sexual harassment occurrence at IHEs. On the other hand, protective factors are proven to reduce the likelihood of incidence of sexual harassment. These factors can exist at different levels of an IHE’s ecology (see Fig. 1): individual, organizational, community, and societal levels (Dahlberg & Krug, 2002). These risk and protective factors can act alone or interact with each other to increase risk for or protect from sexual harassment at IHEs.

Examination of risk and protective factors is integral and preliminary to prevention efforts. Such examination can help identify several points of intervention where we can develop programs to reduce risk and increase prevention. Most of the prior research in this area (Bondestam & Lundqvist, 2020) has been conducted to determine individual risk factors for a traditional undergraduate college population. Research that includes staff and faculty and determines both risk and protective factors at higher levels of an IHE’s ecology is scarce (Bell et al., 2002; Wood et al., 2018). Protective factors that have been associated with lower incidents of sexual harassment at organizations are diverse leadership and its commitment to decreasing sexual harassment, zero tolerance policies, bystander intervention training to prevent and intervene sexual harassment (Mujal et al., 2021) specifically targeting the majority culture (e.g., men), and regular assessment of organizational climate and culture (Bell et al., 2002). The following paragraphs provide a summary of risk factors that have been identified at the four levels of IHE ecology.

At the *individual level*, U.S.-based students, trainees/learners, staff, and faculty who identify as cis-gender women, ethnic minority, sexual minority (LGBQIA), gender minority (transgender, non-binary, genderqueer, non-conforming, or questioning), and who are living with disability are at a higher risk of experiencing sexual harassment (Cantor et al., 2015; Klein & Martin, 2019; Wood et al., 2018). People in the U.S. who are younger in age, spend more time on campus, have insecure employment, consume alcohol in social settings, and have a history of experiencing prior sexual victimization, domestic violence, and bullying are also at a higher risk of experiencing sexual harassment (Abbey, 2011; Campbell et al., 2017; Clear et al., 2014; Clodfelter et al., 2008).

At the *community level*, there is limited research that identifies the risk factors for sexual harassment at.

IHEs. Some U.S.-based studies show that undergraduate students, students attending two-year colleges, students who participate in extracurricular activities, and students involved in sororities and fraternities are at an increased risk of experiencing sexual harassment (American Association of Community Colleges, 2020; Cantor et al., 2019; Howard et al., 2019; Klein & Martin, 2019; Minow & Einolf, 2009). However, it is unknown what contributes to this difference of experiences. Future research highlighting how sexual harassment incidents differ based on the nature of members of IHEs, for example commuter versus residential students, would be beneficial in recognizing the community-level factors and the specific impact of community settings that lead to difference in experiences of sexual harassment for diverse members of IHEs (Howard et al., 2019; Potter et al., 2020).

For prevention at *organizational and systems level*, one must explore organizational aspects such as varied structures, institutions, and inter-relations. Studies from the U.S. show that the environments where sexual harassment is established and normalized are characterized by higher gender-power differentials, unequal gender ratios, hierarchical and dependent structures, contempt and scorn for femineity, culture of silence around sexually harassing behaviors, male-dominated workplaces, isolating learning and training environments, and passive and ineffective leadership (Clancy et al., 2020; Dzau & Johnson, 2018; Ilies et al., 2003). Environments with normalized sexual harassment also tend to have organizational structures that decrease employment engagement, satisfaction, and belongingness at work; are characterized by employment instability; lack transparent communication about discrimination and sexual harassment; and lack investment in efforts to recruit and advance women’s careers and to promote bystander intervention trainings (Bell et al., 2002; Bowes-Sperry & O’Leary-Kelly, 2005).

Further, organizational tolerance of sexual harassment and institutional betrayal have been identified as systems-level trauma-informed risk factors for sexual harassment. Organizational tolerance of sexual harassment includes organization’s failure of making sexual harassment grievances easy to report, taking serious actions against complaints of sexual harassment, sanctioning the perpetrators (Fitzgerald & Cortina, 2018). Institutional betrayal occurs when IHEs cause harm to those dependent on them for protection and safety, for example failure to investigate sexual harassment allegations (Smith & Freyd, 2014).

### Determining the Focus of Prevention: Universal, Selective, and Indicated

Informed by the discipline of public health, prevention programs can have *universal (primary), selective (secondary)*, or *indicated (tertiary)* focus (Gordon, 1983; Institute of Medicine, 1994; Reiss & Price, 1996). Prevention programs with *universal focus* consist of proactive primary prevention strategies to identify the root causes of sexual harassment to ensure its prevention before it begins (Bell et al., 2002). These universal or primary prevention efforts involve efficient and time-limited strategies delivered to all individuals in an organization in large group formats. Examples of existing primary prevention programs include organization-wide trainings during orientation that impart knowledge on the attitudes, beliefs and behaviors that constitute sexual harassment. Such trainings are shown to have only short-term positive effects on participants, but people who participate in these trainings are more prone to identifying sexually harassing behavior than those who do not participate in such trainings. It is important to note that having an awareness of sexually harassing behaviors does not guarantee that an individual will take actions to stop or prevent such behaviors from occurring.

In their systematic review of sexual harassment in higher education, Bondestam and Lundqvist (2020) summarize the characteristics of effective primary prevention sexual harassment programs in IHEs. These characteristics include sensitivity to the sex of the training instructor and to the gender composition of participants; challenging normative assumptions about gender roles; highlighting sexual harassment prevention strategies that are rooted in organizational needs and culture; targeting resistance to changing the organizational culture; directing support from leadership and management in a top-down manner; and using pedagogical methodology to impart knowledge that combines learning in both affective and reflexive ways.

Prevention with a *secondary or selective focus* is designed to target vulnerable populations in IHEs who are at a higher risk of perpetrating or experiencing sexual harassment. Some examples of secondary prevention methods include formal grievance procedures and case management structures that focus on reparations, redressal, and restorative justice for survivors (Koss et al., 2014). There is a lack of evidence of effectiveness of the secondary prevention methods such as case management procedures and formal mechanisms of complaint of sexual harassment in IHEs (Bondestam & Lundqvist, 2020). This lack of evidence is compounded by the data that shows that most sexual harassment incidents go underreported (only 5-30% of all cases are reported), and less than 1% of the reported cases use legal process of redressal (McDonald, 2012). Identifying this challenge, Bondestam and Lundqvist (2020) noted that these compounding challenges have remained intact over the years, hence emphasizing the need for investing in evidence-based secondary prevention methods.

*Indicated or tertiary prevention programs* are designed to target individuals who are either perpetrators or survivors of sexual harassment. Tertiary prevention is closest to after-the-fact intervention strategies and is designed with the aim of mitigating the deleterious consequences of sexual harassment as well as reducing the likelihood of future sexual harassment occurrence, for example restorative programs that focus on reintegrating individuals causing harm back into the community (Koss, 2014). Tertiary prevention programs involve “systematic establishment of accountability for the perpetrators’ own violent actions” (Bondestam & Lundqvist, 2020, p. 408). Although primary prevention is considered the preferred point of intervention for sexual harassment, it may lack the essential dosage and timing to have a long-lasting impact (Weissberg et al., 2003), thus it is essential to also invest in secondary and tertiary prevention programs (Bell et al., 2002).

### Adoption, Adaptation, and Innovation: Pathways Leading to Program Implementation

Once the principles, approach, and focus of prevention have been realized and the risk and protective factors of sexual harassment in the target IHE have been determined, it is crucial to identify the pre-existing evidence-based prevention programs (EBPPs) that can guide the adoption, adaptation, or innovation of a prevention program. A comprehensive review of existing prevention programs guided by the evidence of their effectiveness and their conceptual/practical fit with the target IHE is a good first step in this direction (SAMHSA, 2019). It is also crucial to determine whether the evidence-based program is a good fit with (a) the specific institutional need of the problem (e.g., underreporting, organizational tolerance), (b) the target population at the IHE (e.g., undergraduate students or professional students, students with marginalized backgrounds or intersectional identities), and (c) the type of institution. For example, an EBPP that has evidence of increasing leadership investment in diversity and inclusion and in decreasing organization’s tolerance would be a good conceptual fit for an IHE where systems-level risk factors such as institutional betrayal and lack of transparent communication about discrimination and sexual harassment are the main barriers to sexual harassment prevention.

The result of the comprehensive review to find the best-fit EBPP could lead in one of three directions toward implementation: (1) adopting the program as-is if it represents the best-overall-fit; (2) adapting the program to the target IHE needs and population if it is an imperfect yet viable fit; or (3) developing a new program altogether if no viable pre-existing program is available. An IHE that finds an EBPP that is the best fit for the IHE population, culture, and unique needs can adopt the program. Here, implementation fidelity and effectiveness are key. The adoption of the chosen model should be followed with strict adherence to the original design of the pre-existing program to ensure that it is implemented at the target IHE with fidelity. In real-world settings, however, it is rare to find a pre-existing program that represents a best fit to the target IHE’s unique needs and can be implemented with absolute fidelity. In these cases, carefully planned and executed adaptation can produce desirable outcomes. This requires retaining the core components of the EBPP that are established to be directly responsible for creating positive prevention outcomes. SAMHSA (2019) recommends preserving the setting, maintaining the dosage (e.g., number, length, and frequency of prevention sessions), adding new content as required, and making adaptations with care by working with the original developers of the EBPP and with members of the target IHE. When there is no best-fit or good-fit EBPP for adoption or adaptation, then being innovative and developing a new evidence-informed prevention program may be the best route. This innovation should be guided by existing research in the field and by an assessment of the target IHE population, identifying the optimal culture-based real-world practices to meet the needs of diverse communities and consulting with experts at the local and international levels who can help inform the development of a new prevention program.

In summary, researchers have failed to uncover the root cause of sexual harassment and sexual violence on college and university campuses in the United States. Currently, there is a lack of evidence on who perpetuates sexual harassment, risk and protective factors, prevention programs that work, an identification of factors that are implicated in the effect size (e.g., moderator variables; Linder et al., 2020) culturally responsive implementation and trauma-informed (McCauley & Casler, 2015) methods, and the evaluation and sustainability of prevention and intervention programs. Bonar and colleagues (2022) offer the most comprehensive and inclusive recommendations for prevention science programs for researchers and practitioners to consider. In the context of their recommendations is the importance of ecological validity (e.g., the inclusion of community- and societal-level factors to build multi-level strategies that transform the system and climate that impact and are sustained over time). *A Guide to SAMHSA’S Prevention Strategic Framework* (2019) is a sound option to inform prevention and intervention programs among diverse IHEs (Bonar et al., 2022, Botvin, 2004; Kafonek & Richards, 2017).

There are few empirically supported prevention interventions and programs that effectively target the factors that impact the trajectory, determinants, and short- and intermediate-effects and outcomes of sexual harassment (Bonar et al., 2022; Clancy et al., 2020; Kafonek & Richards, 2017; Walsh et al., 2021) in diverse higher education contexts (e.g., comprehensive universities, Historically Black Colleges, and Universities, community colleges). Many IHEs have a focus on evidence-based prevention practices, policies, and programs (Botvin, 2004). Recommendations for the use (or uptake) of empirically-supported prevention programs ought to be flexible and transportable given the diversity of IHEs and the diverse population who they serve and employ. We contend *A Guide to SAMHSA’S Prevention Strategic Framework* (2019) fits the recommendations proffered by Botvin (2004) and others (e.g., Clancy et al., 2020) and can serve as an exemplar that can be culturally tailored and ecologically valid for diverse IHEs. Another benefit of the proposed adapted *SAMHSA’S Prevention Strategic Framework* (2019) is the recognition of the criticality of systems. Sexual harassment is a systems problem which negatively impacts both the system itself and all of its constituents (Bell et al., 2002). The next section outlines steps to our proposed adapted *SAMHSA’S Prevention Strategic Framework* (2019) that can be used in IHEs and that affords an approach and process that addresses the limitations in the literature base.

## Proposed Framework for Prevention of Sexual Harassment in IHEs

**Fig. 2:**
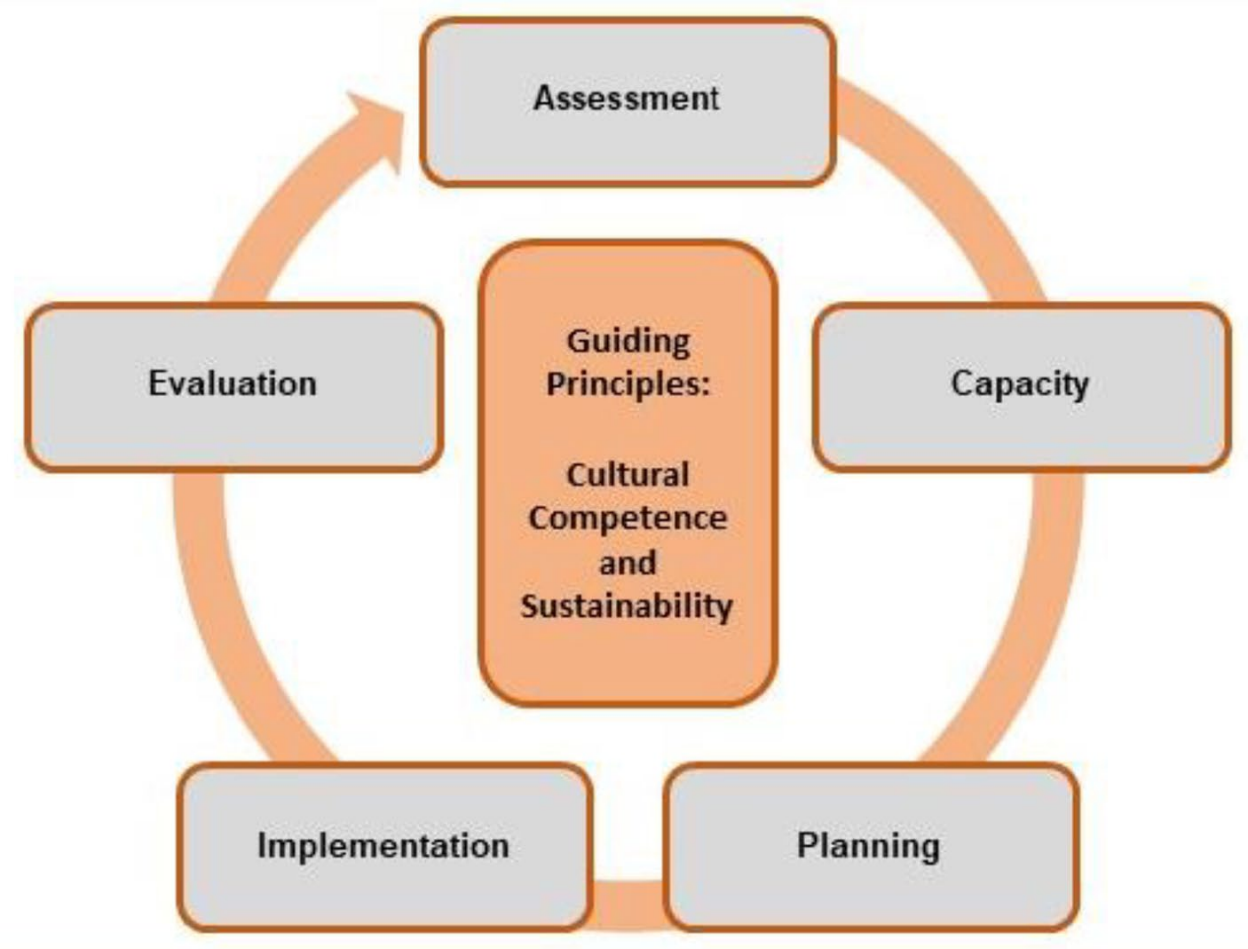
Proposed framework for preventing sexual harassment in higher education. SOURCE: Adapted from Substance Abuse and Mental Health Services Administration (2019)

A framework can be described as a road map that informs the step-by-step process or a prescriptive series of steps that guides how the prevention program should be implemented (Bauer et al., 2015; Meyers et al., 2012). Our proposed framework (see Fig. 2) is adapted from SAMHSA (2019) and is guided by evidence-based prevention principles, focus, approach, and examination of risk and protective factors as described in Table 2. The following paragraphs highlight the five key steps of our proposed framework: assessment, capacity building, planning, implementation, and evaluation. It is important to note that these steps, although presented linearly, can also be implemented iteratively; for example, it may be necessary to return to step 1 (assessment) if the expected outcomes of the prevention program are not achieved.

### Step 1: Assessment

Assessment entails determining the scope of sexual harassment in the target IHE, identifying the vulnerable populations most impacted by sexual harassment, examining risk and protective factors, conducting needs assessment, identifying gaps in existing data, and including previously ignored diverse voices related to sexual harassment. Other key data to gather during the assessment phase include (a) the aspects of sexual harassment that are a priority, (b) the frequency of different types of sexual harassment, (c) the people most vulnerable to experiencing sexual harassment, (d) the key characteristics of the perpetrators of sexual harassment among students, learners, staff, faculty, and administrative leaders, and (e) the magnitude, severity, and trends of sexual harassment. It is also crucial to regularly assess the organizational culture, climate, and context. Organizational culture may include an organization’s languages, attitudes, beliefs, values, and experiences, as well as those of its key stakeholders and target population. Annual institution or department-wide climate surveys that assess individual, community, organizational, and systems level risk and protective factors at an IHE can both direct the priority-based efforts of the prevention program and can demonstrate evidence of effectiveness of the prevention program.

### Step 2: Capacity Building

Capacity building entails determining the extent to which the organization has the necessary infrastructure (e.g., financial and human resources, leadership “buy-in”), knowledge, tools, resources, and trained individuals (i.e., trauma- and equity-informed) to provide the appropriate prevention services (e.g., cultural and linguistic competence and cultural humility training, implicit bias training, and trauma-informed practices). Other critical aspects of the capacity building step are engaging diverse stakeholders, raising organizational awareness about the priority problem, and assessing the organization’s readiness and capacity to adopt, adapt, or develop an effective prevention program based on the strategies described above.

### Step 3: Planning

Planning involves engaging IHEs’ diverse stakeholders and building consensus regarding the priority problems related to sexual harassment that need to be addressed first. Receiving input from and building consensus among diverse stakeholders ensures cultural and ecological validity of the prevention program. A logic model can play a crucial role at this step since it highlights the inputs, activities, resources, outputs, and the outcomes (short-term, intermediate-term, and long-term; Lawton et al., 2014). Incorporation of diverse organizational and community voices and the use of logic model would also help identify if a prevention program needs to be adopted (i.e., use all aspects of a prevention program), adapted (i.e., use the primary aspects of a prevention program and make some changes to culturally fit the organization and population), or developed anew.

### Step 4: Implementation

Implementation entails implementation of the planned prevention program with cultural compatibility, fidelity, and flexibility. Proper implementation ensures successful adaptation of the prevention program to meet the unique needs of an organization and its community. SAMHSA (2019) contends that evidence-based programs are effective when most of the components of empirically supported programs are retained and implemented with fidelity. SAMHSA recommends that adapting pre-existing prevention programs should be advanced with caution and care to culture-fit. Although cultural adaptations may be needed, knowledge experts in the target IHE’s culture should be consulted before implementing adapted programs and cultural adaptations should be documented.

### Step 5: Evaluation

An empirically supported evaluation of the prevention program can help establish its effectiveness and can identify any changes that might improve its implementation. Here, evaluation is defined as “the systematic collection and analysis of information about prevention activities to reduce uncertainty, improve effectiveness, and facilitate decision making” (SAMHSA, 2019, p. 20). This step includes both process and outcome evaluations. Process evaluation determines whether the prevention program activities have been implemented as intended. Outcome evaluation determines the extent to which the prevention program has impacted the outcomes of the program as intended. Both process and outcome evaluation consider the prevention program’s utility, feasibility, propriety, and accuracy in accordance with the desired program outcomes. SAMHSA also recommends that any adaptions made during the adaptation phase be documented (SAMHSA, 2019) and follow-up interviews and data collection regarding the prevention program be conducted to ensure comprehensive and inclusive evaluation.

**Table 2:** Prevention science program development framework. (adapted from SAMHSA, 2019)

Step 1: Assessment	Step 2: Capacity Building	Step 3: Planning	Step 4: Implementation	Step 5: Evaluation and Dissemination
Assess problems and related behaviors	Engage organizational stakeholders	Prioritize protective and risk factors	Deliver programs and practices	Conduct process evaluation
Prioritize problems (magnitude, trends, severity, comparison)	Develop and strengthen a prevention team	Select prevention interventions with empirical support and organizational fit	Balance fidelity with flexibility and necessary adaptations	Conduct outcome evaluation
Assess risk and protective factors	Raise organizational awareness	Develop a plan that is consistent with a logic model	Retain core components	Disseminate evaluation outcomes
Assess available resources	Engage organizational stakeholders	Prioritize protective and risk factors	Establish implementation supports	Make improvements
			Deliver programs and practices	Conduct process evaluation

The dearth of evidence-based sexual harassment prevention programs in IHEs highlights the critical need to develop preventative and protective measures (Walsh et al., 2021). An empirically supported prevention science framework can guide the development of evidence-informed interventions that can help determine stakeholders, outcomes, mechanisms of change, and evaluation protocols for continued improvement. These evaluation protocols will in turn, guide and inform what you do, with whom, how, what you are trying to accomplish at what level, and how you evaluate the intervention. A focus on prevention science-based programs and evaluation will help the field move beyond fragmented solutions which have not been shown to be effective (e.g., sexual harassment grievance procedures, environmental assessment for prevalence of sexual harassment, and compliance with federal legislation), toward comprehensive and sustainable ways of preventing sexual harassment at IHEs (Dobbin & Kalev, 2019; Kafonek & Richards, 2017).

## Conclusion

Sexual harassment is an intractable problem that harms the students, communities, climate/culture, and success of institutes of higher education. Currently, there are few empirically supported prevention interventions and programs that effectively target the factors that impact the trajectory, determinants, and short- and intermediate-effects and outcomes of sexual harassment (Bonar et al., 2022; Clancy et al., 2020; Kafonek & Richards, 2017; Walsh et al., 2021) in diverse higher of education contexts (e.g., comprehensive universities, Historically Black Colleges, and Universities, community colleges). Additionally, many prevention programs and evaluation methods lack rigor, consistency, and an organizing framework (Biglan et al., 2003; Magley et al., 2013). Given the diversity among IHEs, we outlined a framework based on prevention science that can be culturally tailored and scaled up with varied IHEs and diverse stakeholders (e.g., staff, students, faculty, and community members; Wong et al., 2017). Our framework begins with theory from the literature (SAMHSA framework, prevention science, ecological systems) to hypothesize about how to prevent, delay, or minimize the impact of sexual harassment in IHEs. Next, our framework helps to identify the variables (sexual harassment risk and protective factors at multiple levels of an individual’s and system’s ecology) to manipulate and measure through group assignment to understand root causes of sexual harassment and develop interventions to target those root causes. Finally, an empirical study would be conducted based on experimentation to understand how to prevent sexual harassment (i.e., effectiveness of effectiveness of interventions). Findings from the experiment can strengthen and refine sexual harassment prevention theory, which would help us better understand how to prevent or delay sexual harassment or minimize its negative effects. Thus, our framework takes a positivistic ontological approach to science and aligns with the hypothetico-deductive method (Park et al., 2020).

More specifically, this paper proposed an updated, empirically supported organizing framework for the prevention of sexual harassment in IHEs guided by prevention science. The transdisciplinary nature of prevention science is ideal to undergird the prevention of sexual harassment programs in IHEs. Additionally, Botvin (2004) asserted that “culturally competent prevention is the only type of prevention worth doing—and sustaining” (p. 30). We concur with Botvin (2004) that recommendations for prevention programs should be simple, flexible, easy to use, culturally competent, trauma-and-equity-informed, and sustainable. Our framework addresses the spectrum of gender-based violence, including gender-based harassment, the most common form of harassment (Bondestam & Lundqvist, 2020). We also focus on change at multiple levels of IHEs, and marginalized groups, and the need for systematic, on-going evaluation efforts. Our proposed framework places an explicit focus on a limitation described by Kafonek and Richards (2017). They contended few IHEs have prevention programs focused on IHE targeted populations who often are at the greatest risk for harassment (e.g., racial, ethnic, gender, or sexual minority students). An additional benefit of the proposed framework addresses a limitation discussed in the literature: attention to individual- and systemic-level risk and protective factors that ought to be considered in the prevention and intervention efforts of sexual harassment programs in IHEs. Sexual harassment is a systems problem, which Bell and colleagues (2002) described as “dysfunctional organizational behavior…with negative consequences for others in an organization and for the organization itself” (p. 161). Taken together, although we are uniquely focused on IHEs in this paper, it is likely that this framework could be used in other organizations and systems.

Our proposed five-step framework for preventing sexual harassment in IHEs is based on evidence-based prevention principles and approaches. These steps provide the roadmap to targeting sexual harassment prevention at the primary, secondary, and tertiary levels at both the individual and organizational levels embedded in IHEs’ unique socio-cultural contexts. Future research on the application and translation of this framework as well as evidence-based evaluation of prevention programs can help guide researchers, practitioners, IHE leaders, and policymakers toward the most effective sexual harassment prevention programs – their conceptualization, development, implementation, outcomes, and evaluation. There is no doubt that more research is needed to determine what prevention programs—and thus ingredients—work for whom (e.g., perpetrator, survivor, university community), in what context (e.g., type of university, student population, and community characteristics) at what level or levels (Bronfenbrenner, 1976) on short- and long-term outcomes (DeGue et al., 2014).

In conclusion, we recognize that our proposed framework is informed by a dearth of accumulated research specific to IHEs. Marine and Hurtado (2021) contended: “most research conducted on sexual violence and sexual harassment in higher education to date draws data and inferences from problematically homogeneous student samples: White, cisgender, and heterosexual women” (p. 9). On the other hand, the benefit of using prevention science and principles to undergird the proposed framework is a strength. Specifically, the benefit of prevention science in reducing problems, increasing wellness, and promoting positive outcomes across separate and overlapping levels to reduce sexual harassment in IHEs has promise (APA, 2014; Kafonek & Richards, 2017). Because evidence-based prevention policies, programs, and practices to prevent sexual harassment would be consistent with a mission of promoting inclusion, well-being, and a safe environment, IHEs can adapt, adopt, or develop and implement programs based on these guidelines (Botvin, 2004).

## References

[CR1] Abbey, A. (2011). Alcohol’s role in sexual violence perpetration: Theoretical explanations, existing evidence, and future directions. *Drug and Alcohol Review*, *30*, 481–489. 10.1111/j.1465-3362.2011.00296.x 21896070 10.1111/j.1465-3362.2011.00296.x PMC3177166

[CR2] American Association of Community Colleges (2020). *Fast facts 2020* American Association of Community Colleges. https://www.aacc.nche.edu/wp-content/uploads/2020/03/AACC-2020-Fact-Sheet-Final-1_web.jpg

[CR3] American Psychological Association. (2014). Guidelines for prevention in psychology. *American Psychologist*, *69*(3), 285–296. 10.1037/a0034569 24188360 10.1037/a0034569

[CR4] Anderson, L. A., & Whiston, S. C. (2005). Sexual assault education programs: A meta-analytic examination of their effectiveness. *Psychology of Women Quarterly*, *29*(4), 374–388. 10.1111/j.1471-6402.2005.00237.x 10.1111/j.1471-6402.2005.00237.x

[CR5] Aycock, L. M., Hazari, Z., Brewe, E., Clancy, K. B. H., Hodapp, T., & Goertzen, R. M. (2019). Sexual harassment reported by undergraduate female physicists. *Physical Review Physics Education Research*, *15*(010121), 1–13. 10.1103/PhysRevPhysEducRes.15.010121 10.1103/PhysRevPhysEducRes.15.010121

[CR6] Basile, K. C., D’Inverno, A. S., & Wang, J. (2020). National prevalence of sexual violence by a workplace-related perpetrator. *American Journal of Preventive Medicine*, *58*(2), 216–223. 10.1016/j.amepre.2019.09.011 31831292 10.1016/j.amepre.2019.09.011 PMC7092813

[CR7] Bauer, C., Hofer, J., Althaus, H., Duce, A. D., & Simons, A. (2015). The environmental performance of current and future passenger vehicles: Life cycle assessment based on a novel scenario analysis framework. *Applied Energy*, *157*, 871–883. 10.1016/j.apenergy.2015.01.019 10.1016/j.apenergy.2015.01.019

[CR8] Bell, M. P., Quick, J. C., & Cycyota, C. S. (2002). Assessment and prevention of sexual harassment of employees: An applied guide to creating healthy organizations. *International Journal of Selection and Assessment*, *10*(1–2), 160–167. 10.1111/1468-2389.00203 10.1111/1468-2389.00203

[CR9] Biglan, A., Mrazek, P. J., Carnine, D., & Flay, B. R. (2003). The integration of research and practice in the prevention of youth problem behaviors. *American Psychologist*, *58*(6–7), 433–440. 10.1037/0003-066X.58.6-7.433 12971189 10.1037/0003-066X.58.6-7.433

[CR10] Bloom, B. E., Sorin, C. R., Wagman, J. A., & Oaks, L. (2021). Employees, advisees, and emerging scholars: A qualitative analysis of graduate students’ roles and experiences of sexual violence and sexual harassment on college campuses. *Sexuality & Culture*, *25*, 1653–1672. 10.1007/s12119-021-09841-w 34776727 10.1007/s12119-021-09841-w PMC8550674

[CR11] Bonar, E. E., DeGue, S., Abbey, A., Coker, A. L., Lindquist, C. H., McCauley, H. L., Miller, E., Senn, C. Y., Thompson, M. P., Ngo, Q. M., Cunningham, R. M., & Walton, M. A. (2022). Prevention of sexual violence among college students: Current challenges and future directions. *Journal of American College Health*, *70*(2), 575–588. 10.1080/07448481.2020.1757681 32407244 10.1080/07448481.2020.1757681 PMC7666108

[CR12] Bondestam, F., & Lundqvist, M. (2020). Sexual harassment in higher education–a systematic review. *European Journal of Higher Education*, *10*(4), 397–419. 10.1080/21568235.2020.1729833 10.1080/21568235.2020.1729833

[CR13] Botvin, G. J. (2004). Advancing prevention science and practice: Challenges, critical issues, and future directions. *Prevention Science*, *5*(1), 69–72. 10.1023/b:prev.0000013984.83251.8b 15058915 10.1023/b:prev.0000013984.83251.8b

[CR14] Bowes-Sperry, L., & O’Leary-Kelly, A. M. (2005). To act or not to act: The dilemma faced by sexual harassment observers. *The Academy of Management Review*, *30*(2), 288–306. https://www.jstor.org/stable/20159120 10.5465/amr.2005.16387886

[CR15] Bronfenbrenner, U. (1976). The experimental ecology of education. *Teachers College Record*, *78*(2), 1–37. 10.1177/016146817607800201

[CR16] Burn, S. M. (2019). The psychology of sexual harassment. *Teaching of Psychology*, *46*(1), 96–103. 10.1177/0098628318816183 10.1177/0098628318816183

[CR18] Campbell, J. C., Sabri, B., Budhathoki, C., Kaufman, M. R., Alhusen, J., & Decker, M. R. (2017). Unwanted sexual acts among university students: Correlates of victimization and perpetration. *Journal of Interpersonal Violence*, *36*(1–2), NP504–NP526. 10.1177/0886260517734221 29294944 10.1177/0886260517734221 PMC5878971

[CR19] Cantor, D., Fisher, B., Chibnall, S., Harps, S., Townsend, R., Thomas, G., Lee, H., Kranz, V., Herbison, R., & Madden, K. (2019). *Report on the AAU climate survey on sexual assault and sexual misconduct* Westat.

[CR20] Cantor, D., Fisher, B., Chibnall, S., Townsend, R., Lee, H., Bruce, C., & Thomas, G. (2015). *Report on the AAU climate survey on sexual assault and sexual misconduct* Westat.

[CR22] Chambers, K., Romsa, B., & Romsa, K. (2021). Prevention of sexual misconduct on college campuses: A qualitative analysis. *College Student Affairs Journal*, *39*(1), 28–42. 10.1353/csj.2021.0002 10.1353/csj.2021.0002

[CR24] Clancy, K. B. H., Cortina, L. M., & Kirkland, A. R. (2020). Opinion: Use science to stop sexual harassment in higher education. *Proceedings of the National Academy of Sciences of the United States of America*, *117*(37), 22614–22618. 10.1073/pnas.2016164117 32817430 10.1073/pnas.2016164117 PMC7502731

[CR25] Clear, E. R., Coker, A. L., Cook-Craig, P. G., Bush, H. M., Garcia, L. S., Williams, C. M., Lewis, A. M., & Fisher, B. S. (2014). Sexual harassment victimization and perpetration among high school students. *Violence against Women*, *20*(10), 1203–1219. 10.1177/1077801214551287 25288593 10.1177/1077801214551287

[CR26] Clodfelter, T. A., Turner, M. G., Hartman, J. L., & Kuhns, J. B. (2008). Sexual harassment victimization during emerging adulthood. *Crime & Delinquency*, *56*(3), 455–481. 10.1177/0011128708324665 10.1177/0011128708324665

[CR27] Coie, J. D., Watt, N. F., West, S. G., Hawkins, J. D., Asarnow, J. R., Markman, H. J., Ramey, S. L., Shure, M. B., & Long, B. (1993). The science of prevention: A conceptual framework and some directions for a national research program. *American Psychologist*, *48*(10), 1013–1022. 10.1037/0003-066X.48.10.1013 8256874 10.1037/0003-066X.48.10.1013

[CR28] Coulter, R. W. S., Mair, C., Miller, E., Blosnich, J. R., Matthews, D. D., & McCauley, H. L. (2017). Prevalence of past-year sexual assault victimization among undergraduate students: Exploring differences by and intersections of gender identity, sexual identity, and race/ethnicity. *Prevention Science*, *18*, 726–736. 10.1007/s11121-017-0762-8 28210919 10.1007/s11121-017-0762-8 PMC5511765

[CR21] Dahlberg, L. L. & Krug, E. G. (2002). Violence: A Global Public Health Problem. In: Krug, E. G., Mercy, J. A., Dahlberg, L. L., & Zwi, A. B. Lozano R., eds. (2002). *World Report on Violence and Health*. Geneva, Switzerland: World Health Organization; p. 1–21.https://www.cdc.gov/violenceprevention/about/social-ecologicalmodel.html

[CR29] DeGue, S., Valle, L. A., Holt, M. K., Massetti, G. M., Matjasko, J. L., & Tharp, A. T. (2014). A systematic review of primary prevention strategies for sexual violence perpetration. *Aggression and Violent Behavior*, *19*, 346–362. 10.1016/j.avb.2014.05.004 29606897 10.1016/j.avb.2014.05.004 PMC5875446

[CR30] Dills, J., Fowler, D., & Payne, G. (2016). *Sexual violence on campus: Strategies for prevention*. National Center for Injury Prevention and Control, Centers for Disease Control and Prevention. https://www.cdc.gov/violenceprevention/pdf/campussvprevention.pdf

[CR31] Dobbin, F. (2019). A. Kalev (Ed.), The promise and peril of sexual harassment programs. *Proceedings of the National Academy of Sciences* 116 25 12255–12260 10.1073/pnas.1818477116 10.1073/pnas.1818477116 PMC6589754 31160444

[CR32] Dzau, V. J., & Johnson, P. A. (2018). Ending sexual harassment in academic medicine. *New England Journal of Medicine*, *379*, 1589–1591. 10.1056/NEJMp1809846 30207831 10.1056/NEJMp1809846

[CR36] Fitzgerald, L. F., & Cortina, L. M. (2018). Sexual harassment in work organizations: A view from the 21st century. In C. B. Travis, J. W. White, A. Rutherfors, W. S. Williams, S. L. Cook, & K. F. Wyche (Eds.), *APA Handbook of the Psychology of Women: Vol 2. Perspectives on Women’s Private and Public Lives* (pp. 215–234). American Psychological Association. 10.1037/0000060-012

[CR38] Fnais, N., Soobiah, C., Chen, M. H., Lillie, E., Perrier, L., Tashkhandi, M., Straus, S. E., Mamdani, M., Al-Omran, M., & Tricco, A. C. (2014). Harassment and discrimination in medical training: A systematic review and meta-analysis. *Academic Medicine*, *89*(5), 817–827. 10.1097/ACM.0000000000000200 24667512 10.1097/ACM.0000000000000200

[CR39] Goldsmith, R. E., Martin, C. G., & Smith, C. P. (2014). Systemic trauma. *Journal of Trauma & Dissociation*, *15*(2), 117–132. 10.1080/15299732.2014.871666 24617751 10.1080/15299732.2014.871666

[CR40] Gordon, R. S. Jr. (1983). An operational classification of disease prevention. *Public Health Reports*, *98*(2), 107–109. https://www.jstor.org/stable/4627374 6856733 PMC1424415

[CR110] Guilbeau, J., Linder, C., Stewart, D. L. and Sun, J. (2021). *Association for the study of higher education (ASHE) response to the department of education’s May 2020 regulations on title IX of the higher education act of 1972 (Nondiscrimination on the basis of Sex in education programs or activities receiving federal financial assistance).*

[CR43] Howard, R. M., Potter, S. J., Guedj, C. E., & Moynihan, M. M. (2019). Sexual violence victimization among community college students. *Journal of American College Health*, *67*(7), 674–687. 10.1080/07448481.2018.1500474 30257142 10.1080/07448481.2018.1500474

[CR44] Ilies, R., Hauserman, N., Schwochau, S., & Stibal, J. (2003). Reported incidence rates of work-related sexual harassment in the United States: Using meta‐analysis to explain reported rate disparities. *Personnel Psychology*, *56*(3), 607–631. 10.1111/j.1744-6570.2003.tb00752.x 10.1111/j.1744-6570.2003.tb00752.x

[CR45] Institute of Medicine. (1994). *Reducing risks for mental disorders: Frontiers for preventive intervention research*. National Academies Press (US. 10.17226/2139 25144015

[CR47] Kafonek, K., & Richards, T. R. (2017). An examination of strategies for the prevention of gender-based violence at four-year institutions of higher education. *Journal of School Violence*, *16*(3), 271–285. 10.1080/15388220.2017.1318576 10.1080/15388220.2017.1318576

[CR48] Klein, L. B., & Martin, S. L. (2019). Sexual harassment of college and university students: A systematic review. *Trauma Violence & Abuse*, *22*(4), 777–792. 10.1177/1524838019881731 10.1177/1524838019881731 31635552

[CR49] Koss, M. P. (2014). The RESTORE program of restorative justice for sex crimes: Vision, process, and outcomes. *Journal of Interpersonal Violence*, *24*(9), 1623–1660. 10.1177/0886260513511537 10.1177/0886260513511537 24368680

[CR50] Koss, M. P., Wilgus, J. K., & Williamsen, K. M. (2014). Campus sexual misconduct: Restorative justice approaches to enhance compliance with title IX guidance. *Trauma Violence & Abuse*, *15*(3), 242–257. 10.1177/1524838014521500 10.1177/1524838014521500 24776460

[CR52] Lawton, B., Brandon, P. R., Cicchinelli, L., & Kekahio, W. (2014). Logic models: A tool for designing and monitoring program evaluations. (REL 2014–007). Washington, DC: U.S. Department of Education, Institute of Education Sciences, National Center for Education Evaluation and Regional Assistance, Regional Educational Laboratory Pacific. Retrieved from https://ies.ed.gov/ncee/rel/Products/Region/northeast/Publication/3670

[CR53] Linder, C., Grimes, N., Williams, B. M., Lacy, M. C., & Parker, B. (2020). What do we know about campus sexual violence? A content analysis of 10 years of research. *The Review of Higher Education*, *43*(4), 1017–1040. 10.1353/rhe.2020.0029 10.1353/rhe.2020.0029

[CR54] Lisak, D., & Miller, P. M. (2002). Repeat rape and multiple offending among undetected rapists. *Violence and Victims*, *17*(1), 73–84. 11991158 10.1891/vivi.17.1.73.33638

[CR55] Magley, V. J., Fitzgerald, L. F., Salisbury, J., Drasgow, F., & Zickar, M. J. (2013). Changing sexual harassment within organizations via training interventions: Suggestions and empirical data. In C.L. Cooper (Ed.), *The Fulfilling Workplace*, (1st ed., pp. 245–266). Routledge. 10.4324/9781315557953

[CR56] Marine, S. B., & Hurtado, S. S. (2021). Association for the Study of Higher Education (ASHE) response to the Department of Education’s May 2020 regulations on Title IX of the Higher Education Act of 1972 (Nondiscrimination on the Basis of Sex in Education Programs or Activities Receiving Federal Financial Assistance). *Association for the Study of Higher Education*, 1–43. 2021.03 ASHE Response to ED’s May 2020 regulations on Title IX of the Higher Education Act of 1972.pdf.

[CR57] McCauley, H. L., & Casler, A. W. (2015). College sexual assault: A call for trauma-informed prevention. *The Journal of Adolescent Health*, *56*(6), 584–585. 10.1016/j.jadohealth.2015.03.012 26003573 10.1016/j.jadohealth.2015.03.012 PMC4737642

[CR58] McDonald, P. (2012). Workplace sexual harassment 30 years on: A review of the literature. *International Journal of Management Reviews*, *14*, 1–17. 10.1111/j.1468-2370.2011.00300.x 10.1111/j.1468-2370.2011.00300.x

[CR60] Meyers, D. C., Durlak, J. A., & Wandersman, A. (2012). The quality implementation framework: A synthesis of critical steps in the implementation process. *American Journal of Community Psychology*, *50*, 462–480. 10.1007/s10464-012-9522-x 22644083 10.1007/s10464-012-9522-x

[CR61] Minow, J. C., & Einolf, C. J. (2009). Sorority participation and sexual assault risk. *Violence against Women*, *15*(7), 835–851. 10.1177/1077801209334472 19458092 10.1177/1077801209334472

[CR62] Mujal, G. N., Taylor, M. E., Fry, J. L., Gochez-Kerr, T. H., & Weaver, N. L. (2021). A systematic review of bystander interventions for the prevention of sexual violence. *Trauma Violence & Abuse*, *22*(2), 381–396. 10.1177/1524838019849587 10.1177/1524838019849587 31204606

[CR63] Nation, M., Crusto, C., Wandersman, A., Kumpfer, K. L., Seybolt, D., Morrissey-Kane, E., & Davino, K. (2003). What works in prevention: Principles of effective prevention programs. *American Psychologist*, *58*(6–7), 449–456. 10.1037/0003-066X.58.6-7.449 12971191 10.1037/0003-066X.58.6-7.449

[CR64] National Academies of Sciences, Engineering, and Medicine (2018). Sexual harassment of women: climate, culture, and consequences in academic sciences, engineering, and medicine. The National Academies Press. 10.17226/24994 29894119

[CR65] Park, Y., Konge, L., & Artino, A. R. (2020). The positivism paradigm of research. *Academic Medicine: Journal of the Association of American Medical Colleges*, *95*(5). 10.1097/ACM.0000000000003093 10.1097/ACM.0000000000003093 31789841

[CR68] Potter, S. J., Fox, N., Smith, D., Draper, N., Moschella, E. A., & Moynihan, M. M. (2020). Sexual assault prevalence and community college students: Challenges and promising practices. *Health Education & Behavior*, *47*(15), 7S–16S. 10.1177/1090198120910988 32250186 10.1177/1090198120910988

[CR69] Reiss, D., & Price, R. H. (1996). National research agenda for prevention research: The national institute of mental health report. *American Psychologist*, *51*(11), 1109–1115. 8937258 10.1037/0003-066X.51.11.1109

[CR71] Shapiro, V. B., Eldeeb, N., McCoy, H., Trujillo, M., & Jones, T. M. (2024). Where’s the BIPOC blueprint for healthy youth development? The role of scientific omissions in our struggle for science translation and racial equity in the United States. *Journal of Prevention*, 1–19. 10.1007/s10935-024-00771-5 PMC10981621 38353805

[CR72] Smith, C. P., & Freyd, J. J. (2014). Institutional betrayal. *American Psychologist*, *69*(6), 575–587. 10.1037/a0037564 25197837 10.1037/a0037564

[CR74] Substance Abuse and Mental Health Services Administration [SAMHSA] (2014). *SAMHSA’s concept of trauma and guidance for a trauma-informed approach*. (HHS Publication No. (SMA) 14-4884). SAMHSA’s Concept of Trauma and Guidance for a Trauma-Informed Approach (hhs.gov).

[CR75] Substance Abuse and Mental Health Services Administration (2019). *A Guide to SAMHSA’s strategic prevention framework* https://www.samhsa.gov/sites/default/files/20190620-samhsa-strategic-prevention-framework-guide.pdf

[CR76] Swedish Council of Higher Education (2020). *Efforts to prevent sexual harassment in academia. An international research review*. (1). https://www.uhr.se/globalassets/_uhr.se/publikationer/2020/uhr-efforts-to-prevent-sexual-harassment-in-academia.pdf

[CR77] Vladutiu, C. J., Martin, S. L., & Macy, R. J. (2011). College- or university-based sexual assault prevention programs: A review of program outcomes, characteristics, and recommendations. *Trauma Violence & Abuse*, *12*(2), 67–86. 10.1177/1524838010390708 10.1177/1524838010390708 21196436

[CR78] Walsh, K., Sarvet, A. L., Khan, S., Choo, T., Wall, M., Santelli, J., Wilson, P., Gilbert, L., Reardon, L., Hirsch, J. S., & Mellins, C. A. (2021). Socio-ecologically constituted types of sexual assault. *Psychology of Women Quarterly*, *45*(1), 8–19. 10.1177/0361684320964452 10.1177/0361684320964452

[CR81] Weissberg, R. P., Kumpfer, K. L., & Seligman, M. E. P. (2003). Prevention that works for children and youth: An introduction. *American Psychologist*, *58*(6–7), 425–432. 10.1037/0003-066X.58.6-7.425 12971188 10.1037/0003-066X.58.6-7.425

[CR82] Wong, Y. J., Vaughan, E. L., & Klann, E. M. (2017). The science and practice of prevention from multicultural and social justice perspectives. In M. Israelashvili, & J. L. Romano (Eds.), *The Cambridge handbook of international prevention science* (pp. 107–132). Cambridge University Press. 10.1017/9781316104453.007

[CR83] Wood, L., Hoefer, S., Kammer-Kerwick, M., Parra-Cordona, J. R., & Busch-Armendariz, N. (2018). Sexual harassment at institutions of higher education: Prevalence, risk, and extent. *Journal of Interpersonal Violence*, 1–25. 10.1177/0886260518791228 10.1177/0886260518791228 PMC10676016 30071790

